# Acute In Vivo Administration of Compound 21 Stimulates Akt and ERK1/2 Phosphorylation in Mouse Heart and Adipose Tissue

**DOI:** 10.3390/ijms242316839

**Published:** 2023-11-28

**Authors:** Diego T. Quiroga, Jorge A. Narvaéz Pardo, María G. Zubiría, Benjamín Barrales, Marina C. Muñoz, Andrés Giovambattista, Fernando P. Dominici

**Affiliations:** 1 Facultad de Farmacia y Bioquímica, Departamento de Química Biológica and IQUIFIB (UBA-CONICET), Universidad de Buenos Aires, Buenos Aires C1113AAD, Argentina; 2 Laboratorio de Neuroendocrinología, Instituto Multidisciplinario de Biología Celular (IMBICE), CICPBA-CONICET-UNLP), La Plata B1906APO, Argentina

**Keywords:** Akt, AT_2_ receptor, C21, ERK1/2, signaling

## Abstract

The angiotensin II type 2 (AT_2_) receptor has a role in promoting insulin sensitivity. However, the mechanisms underlying the AT_2_ receptor-induced facilitation of insulin are still not completely understood. Therefore, we investigated whether acute in vivo administration of AT_2_ receptor agonist compound 21 (C21) could activate insulin signaling molecules in insulin-target tissues. We report that, in male C57BL/6 mice, an acute (5 min, 0.25 mg/kg; i.v.) injection of C21 induces the phosphorylation of Akt and ERK1/2 at activating residues (Ser473 and Thr202/Tyr204, respectively) in both epididymal white adipose tissue (WAT) and heart tissue. In WAT, the extent of phosphorylation (p) of Akt and ERK1/2 induced by C21 was approximately 65% of the level detected after a bolus injection of a dose of insulin known to induce maximal activation of the insulin receptor (IR). In the heart, C21 stimulated p-Akt to a lesser extent than in WAT and stimulated p-ERK1/2 to similar levels to those attained by insulin administration. C21 did not modify p-IR levels in either tissue. We conclude that in vivo injection of the AT_2_ receptor agonist C21 activates Akt and ERK1/2 through a mechanism that does not involve the IR, indicating the participation of these enzymes in AT_2_R-mediated signaling.

## 1. Introduction

The renin-angiotensin system modulates insulin action mainly through the actions of its principal peptide angiotensin (Ang) II acting on its two subtypes of receptors, angiotensin type 1 receptor (AT_1_R) and angiotensin type 2 receptor (AT_2_R), which belong to the G protein-coupled receptor (GPCRs) family [1,2,3,4,5]. Conditions of chronic elevation of Ang II are associated with insulin resistance. This negative effect is mediated by the AT_1_R. Inhibition of Ang II action through an AT_1_ receptor blockade with specific antagonists or reduction of its production through angiotensin converting enzyme inhibitors results in improvement of glucose homeostasis both in animal models of insulin resistance and/or type 2 diabetes [1,2,3,4,5]. In the last decade, it has been established that the AT_2_R exerts a positive effect on insulin sensitivity [6,7]. In general, targeting the AT_2_R with pharmacological tools clearly supports a favorable role in glucose metabolism and insulin function. This particularly applies to adipose tissue [6,7]. Pharmacological acute antagonism of the AT_2_R with the non-peptide antagonist PD123319 decreased glucose uptake and reduced Akt phosphorylation in rat skeletal muscle [8,9], while chronic blockade of the AT_2_R reduced insulin receptor signaling in terms of PI3K/Akt activation in the liver and adipose tissue [10], suggesting a physiological role for the AT_2_R. Stimulation of the AT_2_R using the established AT_2_R agonist C21 has been associated with improved insulin sensitivity in KK-Ay type 2 diabetic mice [11], in rats fed a high-fat/high-fructose diet [12], in healthy and streptozotocin (STZ)-diabetic rats and mice [13,14,15], in neonatal STZ-diabetic rats [16], in mice with high-fat diet (HFD)-induced obesity [17,18], in healthy, normal C57BL/6 mice [19] and in female diabetic db/db mice [20]. This physiological role of the AT_2_R was also corroborated by a study in AT_2_R-knockout (KO) mice, which showed that in these animals displayed higher STZ-induced glycemia coupled with lower pancreatic insulin levels [15]. However, overall, data from AT_2_R-KO are controversial and support a beneficial role only in female animals [21,22]. Despite this large amount of evidence for favorable metabolic effects exerted by the AT_2_R, the mechanisms by which these effects proceed are not known.

AT_2_R-induced intracellular signaling is atypical and different from the traditional modes of signaling displayed by many other GPCRs including the AT_1_R [6,7]. Initial AT_2_R signaling involves the association of an inhibitory G-protein (Gi) or AT_2_R-interacting protein (ATIP) with the AT_2_R [7]. These early associations lead to subsequent signaling via phosphatase, kinase, and PPARγ pathways. There is strong evidence for the involvement of kinases in the intermediate signaling of the AT_2_R [6,7]. In human aortic endothelial cells, incubation with C21 has been shown to induce a rapid phosphorylation of Akt and ERK1/2 at activating residues indicating a recruitment of these kinases by the AT_2_R [23,24]. There is evidence for the participation of Akt in AT_2_R-induced effects including improvement of insulin signaling [19,20], nitric oxide (NO) production [23,25], adipose fat browning [26], proximal tubule albumin endocytosis [27], osmotic cellular resistance [28] and antiproteinuric actions [29]. Participation of ERK1/2 has been reported in various AT_2_R-mediated actions such as neuronal differentiation [30], skeletal muscle regeneration [31] and eNOS-mediated vasodilation [32]. However, direct AT_2_R-mediated activation of either Akt or ERK1/2 has not been evidenced in vivo yet. Thus, the goal of the current work was to determine whether acute intravenous administration of the AT_2_R agonist C21 could result in phosphorylation of Akt and ERK1/2 in the metabolic tissues of the mouse in vivo. Our results extend the knowledge of the signaling pathways mediated by the AT_2_R and indicate that in vivo injection of C21 induces the activation of both Akt and ERK in mouse white adipose tissue (WAT) and heart tissue. These findings highlight the importance of these two kinases in AT_2_R-mediated signaling.

## 2. Results

### 2.1. C21 Induces the Phosphorylation of Akt and ERK1/2 in Mouse White Adipose Tissue (WAT)

**Figure 1:**
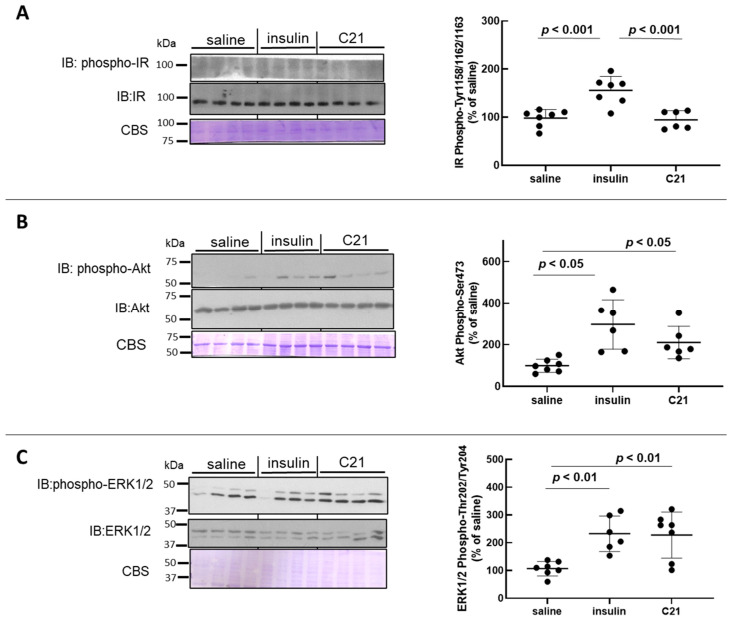
C21 stimulates the phosphorylation of Akt and ERK1/2 in mouse adipose tissue. The phosphorylation level and total abundance of the insulin receptor (IR) (**A**), Akt (**B**) and ERK1/2 (**C**) were evaluated in epididymal adipose tissue homogenates by Western blot. Western blot membranes were stained with Coomassie Blue for loading control. The phosphorylation-to-protein ratio was calculated for each sample. Data are expressed as mean ± SEM (n = 6 for all groups). A representative image is presented. All analyses were carried out using GraphPad Prism 8.0.

For comparison, samples from C21-injected mice were run together with samples of WAT homogenates obtained from insulin (a known recruiter of both Akt and ERK1/2) or vehicle (saline)-injected animals. As compared to baseline values, a bolus injection of insulin known to attain maximal stimulation of the insulin receptor (IR) induced a significant increase in the phosphorylation of the IR at activating Tyr residues (Tyr1158/1162/1163) in WAT (1.6-fold increase; Figure 1A). Accordingly, phospho (p)-Akt-Ser473 levels and p-ERK1/2-Thr202/Tyr204 levels in WAT increased significantly after insulin injection (Figure 1B,C). While acute intravenous injection of C21 did not modify IR phosphorylation in WAT (Figure 1A), acute C21 injection induced a marked and significant increase in both p-Akt and p-ERK1/2 in mouse WAT (Figure 1B,C). The mean level of Akt phosphorylation attained 5 min after C21 administration was approximately 65% of that detected after insulin injection (Figure 1B), while the level of ERK1/2 phosphorylation was comparable to that induced by in vivo insulin administration (Figure 1C). The protein abundance of IR, Akt and ERK1/2 in WAT was not modified after either treatment with saline, insulin or C21 (Figure 1A–C).

### 2.2. C21 Induces the Phosphorylation of Akt and ERK1/2 in Mouse Heart

**Figure 2:**
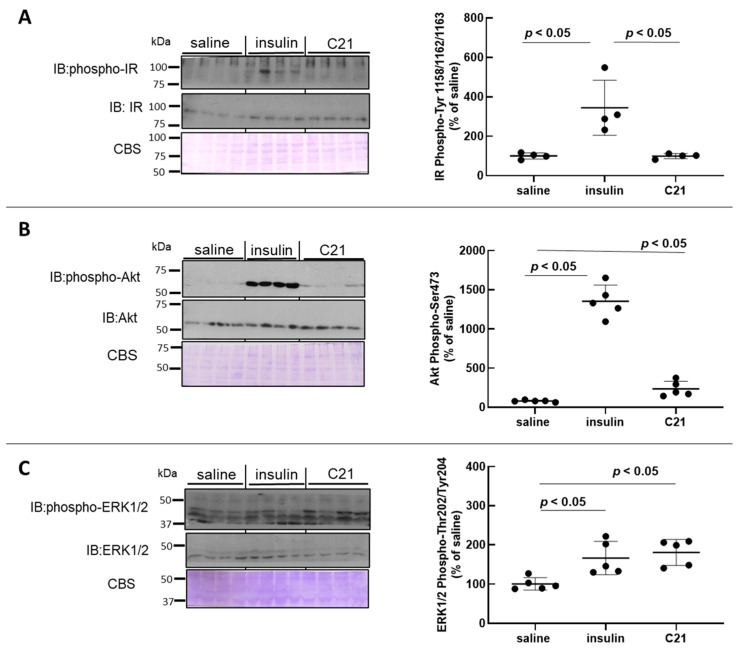
C21 stimulates the phosphorylation of Akt and ERK1/2 in mouse heart. The phosphorylation level and total abundance of the insulin receptor (IR) (**A**), Akt (**B**) and ERK1/2 (**C**) were evaluated in heart homogenates by Western blot. Western blot membranes were stained with Coomassie Blue for loading control. The phosphorylation-to-protein ratio was calculated for each sample. Data are expressed as mean ± SEM (n = 6 for all groups). All analyses were carried out using GraphPad Prism 8.0.

As compared to baseline values, in vivo intravenous injection of insulin induced an approximate 3.5-fold increase in IR phosphorylation at activating residues Tyr1158/1162/1163 (Figure 2A) while, as expected, C21 did not modify IR phosphorylation in mouse heart tissue (Figure 2A).

Similarly to what was detected for WAT, in vivo C21 injection stimulated the phosphorylation levels of Akt at Ser473 by approximately 2.5–3-fold in heart tissue (Figure 2B). However, this stimulation was only a fraction of that attained after insulin administration using the same protocol. When heart homogenates were probed with an anti p-ERK1/2-Thr202/Tyr204 antibody, an approximate 1.8-fold increase over baseline values was detected for ERK1/2 phosphorylation in heart tissue after in vivo C21 injection (Figure 2C). The mean level of Akt phosphorylation attained 5 min after C21 administration was approximately 25% of that detected after insulin injection (Figure 2B), while the level of ERK1/2 phosphorylation was comparable to that induced by in vivo insulin administration (Figure 2C). The protein abundance of IR, Akt and ERK1/2 in heart tissue was not modified after either treatment with saline, insulin or C21 (Figure 2A–C).

## 3. Discussion

The AT_2_R is one of the main receptors within the protective arm of the RAS, others being MAS and insulin-regulated aminopeptidase [6,7]. Compared to other GPCRs of therapeutic significance, the development of drugs targeting the AT_2_R for therapeutic use of its protective and regenerative properties has been slow [6]. The difficulty in determining robust parameters for the detection of AT_2_R effects is likely a major reason for this delay. Since the signaling pathways afford AT_2_Rs the ability to exert protective actions in multiple disease states—sometimes in direct opposition to deleterious AT_1_R-mediated effects—the investigation of these pathways is a topic of importance. Recent reports have reinforced the notion that in vivo stimulation of the AT_2_R with C21 leads to major beneficial actions, including reduction of inflammation [33], attenuation of cardiac fibrosis [34], antagonism of the thromboxane receptor [35], enhancement of insulin sensitivity and amelioration of type-2 diabetes complications [6,19,20].

Intracellular signaling induced by the AT_2_R is atypical and remarkably it does not share a resemblance with traditional modes of signaling displayed by many other GPCRs, including the AT_1_R [6,35,36]. There is evidence that AT_2_R signaling events include the participation of phosphatases, kinases and PPAR pathways. In addition, accumulated evidence indicates that there is a large variety of AT_2_R-stimulated signal transduction pathways, with evidence for both G-protein-dependent and independent mechanisms, a common pattern for GPCRs [6,37,38]. Activation of protein phosphatases is a central intermediate step in AT_2_R signaling, regardless of whether the upstream signaling involves G-proteins or not [6,7,39].

While the signaling pathways employed by the AT_2_R have been the focus of intense research efforts, the role of downstream kinase and phosphatase pathways on AT_2_R-mediated actions requires further investigation. Our results are indicative of the participation of both Akt and ERK1/2 in AT_2_R signaling in both white adipose tissue and heart tissue—tissues known to express the AT_2_R [6,7]. These results are in good agreement with previous reports indicating that stimulation of the AT_2_R using C21 induces Akt phosphorylation in human aortic endothelial cells (HAECs), an event that was linked to NO production [23]. More recently, the phosphorylation status of HAECs after stimulation with C21 was determined utilizing time-resolved quantitative phosphoproteomics, showing that AT_2_Rs stimulation induces the phosphorylation and dephosphorylation of 172 proteins, of which, a large proportion are involved in antiproliferation and apoptosis [24]. Computer-based kinase prediction found that both Akt and ERK1/2 take part in AT_2_R-signaling. Participation of these kinases in AT_2_R-mediated signaling in HAECs was confirmed by Western Blotting [39]. Our current findings are in excellent correlation with this study and indicate that these events also take place in vivo and thus they could be of physiological relevance. At present, it is, however, not known how the connection between the AT_2_R and these downstream kinases proceeds. Unlike most other GPCRs, the AT_2_R does not associate with β-arrestin [40]. Since physical interaction of the AT_2_R with other receptors such as AT_1_R, B_2_R and Mas and with several other binding proteins has been established [41], we hypothesize that these interactions could be relevant for current findings.

Of note, current results support the participation of the kinases Akt and ERK1/2 that has been reported in several AT_2_R-mediated actions such as improvement of insulin signaling [19,20], NO synthesis [23,24], adipose fat browning [25], proximal tubule albumin endocytosis [26], osmotic cellular resistance [27], antiproteinuric actions [28] and anti-fibrotic effects [37], for Akt, and neuronal differentiation [29] skeletal muscle regeneration [30], endothelial NO synthase-mediated vasodilation [31] and mitogen-activated protein kinase phosphatase activation [39], in the case of ERK1/2. Our previous reports involving pharmacological agonism or blockade of the AT_2_R and mice with global deletion of the AT_2_R [10,19,20,22] suggested that the presence of the AT_2_R in adipose tissue is critical to the role of this receptor in the control of insulin action and glucose homeostasis. Considering current findings, it is hypothesized that the kinases Akt and ERK1/2, known to participate in the control of metabolism, could have a role in AT_2_R-mediated metabolic actions in this tissue.

When analyzing the strengths of the study, we considered the following aspects: (a) results contribute to expanding the knowledge of AT_2_R-mediated signaling pathways, strongly supporting the participation of kinases aside from phosphatases; (b) the detection of Akt and ERK1/2 phosphorylation in mouse tissues through the use of phospho-specific antibodies make the results unequivocal; and (c) reported results are ascribed to AT_2_R agonism since C21 is a compound with proven specificity towards this receptor. Noteworthily, it must be mentioned that this study has several limitations. Namely: (a) the utilization of a single species, a single gender and a single dose of C21 at a one-time point is not enough to fully characterize the selectivity and efficacy of the in vivo activation of the analyzed kinases [42]; (b) analysis of Akt and ERK1/2 phosphorylation after co-infusion of C21 with an AT_2_R antagonist would be important to further corroborate that activation of the studied kinases is AT_2_R-mediated; (c) it would be of value to demonstrate that the actions originated by stimulation with C21 are not present in cells in which the AT_2_R is either absent or silenced.

In conclusion, current findings provide new information that contributes to the knowledge of AT_2_R-signaling, by the identification of functional AT_2_Rs in mouse adipose tissue and heart tissue and the demonstration of Akt and ERK1/2 phosphorylation upon in vivo activation of AT_2_Rs in these tissues.

## 4. Materials and Methods

### 4.1. Experimental Animals

All experiments were approved by the Institutional Animal Care and Use Committee of the School of Pharmacy and Biochemistry of the University of Buenos Aires. Adult (3–4 months old) C57BL/6 male mice were used. Animals were housed 3–5 per cage in a room with controlled light (12 h light: 12 h darkness cycle) and temperature (22 ± 2 °C). Mice had free access to a nutritionally balanced diet and tap water.

### 4.2. In Vivo Administration of C21and Tissue Collection

Compound 21 was obtained through Vicore Pharma AB (Göteborg, Sweden). The dose of C21 was calculated based on previous studies aimed at exploring vasodilation or insulin enhancement effects derived from in vivo AT_2_R stimulation [43,44]. With a molecular weight of 475.63 g/mol and the assumption of a blood volume of 1.8 mL in a 20-g mouse [45], the maximal blood concentration of C21 attained immediately after injection would be in the range of 8–10 µM, assuming that no degradation occurred during the timeframe of the experiment. At this concentration, C21 has been shown to evoke vasodilation and to facilitate insulin delivery to tissues [43,44]. The duration of the treatment was selected from previously published studies [23,24].

### 4.3. Western Blot

Western blotting procedures used in this study have been reported previously [19,20]. Information on all antibodies used is presented in Table S1. Adipose tissue and heart extracts were denatured, resolved by SDS-PAGE, transferred into PVDF membranes (Millipore Immobilon-FL; EMD Millipore, Billerica, MA, USA) and finally probed with specific antibodies: anti-phospho-Tyr 1158/1162/1163 insulin receptor β subunit (Millipore, Burlington, MA, USA), IR β subunit (GeneTex, Irvine, CA, USA), Akt, phospho-Ser 473 Akt, ERK1/2 or phospho-Thr202/Tyr204 ERK1/2 (Cell Signaling, Danvers, MA, USA). Immunoreactive bands were detected by chemiluminescence (Pierce™ ECL Plus Western Blotting Substrate, Thermo Fisher Scientific, Waltham, MA, USA). Protein loading control was performed by relativizing protein content to Coomassie Blue staining of PVDF membranes after blotting experiments as previously described [46]. The level of each protein evaluated was normalized to the area obtained from control samples to avoid sources of variation. Phosphorylation values were then related to calculated protein values for each protein analyzed (IR, Akt and ERK1/2). To assess the error of the control group, each individual control value was divided by average intensity obtained for the control group (saline-injected mice). The units shown in bar graphs were obtained by considering the average value of intensity of each specific band in the control group as 100% ± S.E.M). The molecular weight of proteins was estimated using pre-stained protein markers (Bio-Rad, Hercules, CA, USA).

### 4.4. Statistical Analysis

Data are presented as mean ± SEM. Comparisons were performed via one-way ANOVA with the post-hoc Tukey method for multiple groups using Prism software 8.0 (GraphPad, San Diego, CA, USA). Differences were considered statistically significant at *p* < 0.05.

## Data Availability

Data is contained within the article and Supplementary Material.

## Supplementary Materials

The supporting information can be downloaded at: https://www.mdpi.com/article/10.3390/ijms242316839/s1.

## Abbreviations

Ang	Angiotensin
AT_1_R	Angiotensin II receptor type 1
AT_2_R	Angiotensin II receptor type 2
ATIP	AT_2_R-interacting protein
C21	Compound 21
GPCR	G protein-coupled receptor
IR	Insulin receptor
KO	Knockout
NO	Nitric oxide
PPAR	Peroxisome proliferator-activated receptor
NO	Nitric oxide
STZ	Streptozotocin
WAT	White adipose tissue
