# Supplementary material for: Acute In Vivo Administration of Compound 21 Stimulates Akt and ERK1/2 Phosphorylation in Mouse Heart and Adipose Tissue
Source: Int J Mol Sci. 2023 Nov 28;24(23):16839. doi: 10.3390/ijms242316839 (PMC10706736; doi:10.3390/ijms242316839)
Supplement: Supplementary file 1 [file ijms-24-16839-s001.zip › ijms-2718898-supplementary.pdf]

Table S1. List of antibodies.

Name	Source	Catalog #
Phospho-IR/IGF1R Tyr1158/Tyr1162/Tyr1163	Millipore	07-841
IR β subunit	GeneTex	GTX101136
Phospho-Ser 473 Akt	Cell Signaling Technology	4060
Akt	Cell Signaling Technology	4691
Phospho-Thr202/Tyr204 ERK1/2	Cell Signaling Technology	4370
ERK1/2	Cell Signaling Technology	9102
